# Genome-wide identification of *Glycyrrhiza uralensis* Fisch. MAPK gene family and expression analysis under salt stress relieved by *Bacillus subtilis*

**DOI:** 10.3389/fgene.2024.1442277

**Published:** 2024-07-26

**Authors:** Pengchao Gao, Jiancai Xiao, Wanying Guo, Rui Fan, Yan Zhang, Tiegui Nan

**Affiliations:** State Key Laboratory for Quality Ensurance and Sustainable Use of Dao-di Herbs, National Resource Center for Chinese Materia Medica, China Academy of Chinese Medical Sciences, Beijing, China

**Keywords:** *Glycyrrhiza uralensis* Fisch., salt stress, MAPK, *Bacillus subtilis*, subcellular localization

## Abstract

**Introduction:** Research on *Glycyrrhiza uralensis*, a nonhalophyte that thrives in saline–alkaline soil and a traditional Chinese medicinal component, is focused on improving its ability to tolerate salt stress to increase its productivity and preserve its “Dao-di” characteristics. Furthermore, the inoculation of bioagents such as *Bacillus subtilis* to increase plant responses to abiotic stressors is currently a mainstream strategy. Mitogen-activated protein kinase (MAPK), a highly conserved protein kinase, plays a significant role in plant responses to various abiotic stress pathways.

**Methods:** This investigation involved the identification of 21 members of the *GuMAPK* family from the genome of *G*. *uralensis*, with an analysis of their protein conserved domains, gene structures, evolutionary relationships, and phosphorylation sites using bioinformatics tools.

**Results:** Systematic evolutionary analysis of the 21 *GuMAPKs* classified them into four distinct subgroups, revealing significant differences in gene structure and exon numbers. Collinearity analysis highlighted the crucial role of segmental duplication in expanding the *GuMAPK* gene family, which is particularly evident in *G. uralensis* and shows a close phylogenetic relationship with *Arabidopsis thaliana*, tomato, and cucumber. Additionally, the identification of phosphorylation sites suggests a strong correlation between *GuMAPK* and various physiological processes, including hormonal responses, stress resistance, and growth and development. Protein interaction analysis further supported the role of *GuMAPK* proteins in regulating essential downstream genes. Through examination of transcriptome expression patterns, *GuMAPK16-2* emerged as a prospective pivotal regulatory factor in the context of salt stress and *B. subtilis* inoculation, a finding supported by its subcellular localization within the nucleus.

**Discussion:** These discoveries offer compelling evidence for the involvement of GuMAPK in the salt stress response and for the exploration of the mechanisms underlying *B. subtilis*’ enhancement of salt tolerance in *G. uralensis*.

## 1 Introduction

*Glycyrrhiza uralensis* Fisch. is a perennial herbaceous plant of the Fabaceae family, and it is the most extensively used medicinal herb in China, earning the title of “King of Herbs” (Zhang and Ye, 2009; Yan et al., 2023). *G. uralensis* grows mainly in arid or semiarid desert areas, sandy riverbanks, and saline-alkaline soils at altitudes ranging from 400 to 2,700 m and exhibits high ecological adaptability, salt tolerance, and drought resistance. Its moderate salt tolerance contributes significantly to its authenticity as a medicinal herb, maintaining important quality characteristics (Egamberdieva et al., 2021; Shen et al., 2022). Optimal salt concentrations can activate the antioxidative system, calcium ion channels, and hormone (abscisic acid, ethylene) signaling pathways of *G. uralensis*, promoting sodium excretion and ion distribution to increase plant resilience (Li et al., 2024). However, *G. uralensis* is not tolerant to saline–alkaline conditions and cannot thrive in highly saline soils. Exposure to elevated salt levels can result in leaf chlorosis, root mortality due to disrupted water balance, ion toxicity, oxidative stress, and nutritional imbalances in the roots. As saline–alkaline land continues to expand and the demand for *G. uralensis* continues to grow, the practical challenge of insufficient resources has made enhancing its tolerance to saline–alkaline environments a pressing issue in the cultivation of *G. uralensis*.

Plants frequently encounter diverse abiotic stresses and pathogen infections throughout their growth and development. As a result, over the course of long-term evolutionary processes, plants have developed intricate regulatory networks that contribute to both growth and development and to responses to adversities. MAPK is a serine‒threonine protein kinase that is an important part of the MAPK cascade signaling pathway (Jagodzik et al., 2018). Through sequential phosphorylation, MAPK, MAPKK, and MAPKKK together form a highly conserved cascade signal transduction pathway (Zhang and Klessig, 2001; Nakagami et al., 2005; Danquah et al., 2014). The activity of MAPK is believed to be regulated by dual phosphorylation sites in the activation loop amino acid sequence, where phosphorylation signals are transmitted from MAPKKK to MAPK. When MAPKKK is activated by sensors or receptors and phosphorylated, it activates downstream of MAPKK, which in turn activates MAPK through dual phosphorylation of conserved tyrosine and threonine residues in the activation loop (T-LOOP) located between kinase subdomains VII and VIII. Phosphorylation of these sites increases MAPK activity by more than a thousand fold (Ichimura et al., 2002). Previous research has shown the importance and mechanisms of MAPK in coping with salt stress. There is a close relationship between ROS signal transduction and MAPK activation in plants, where H_2_O_2_ acts as a key signaling molecule that can induce the MAPK cascade to activate MEKK1-MKK4/5-MPK3/6 to cope with salt stress (Canagarajah et al., 1997; Jalmi and Sinha, 2015; Sachdev et al., 2021). Given the potential application of MAPK in enhancing crop salt tolerance and stress resistance through genetic improvements, more attention is shifting from traditional cultivation techniques to bioinformatics and functional verification research in abiotic stress biology.

Since Stafstrom et al. (1993) first cloned the first higher plant MAPK protein kinase encoding gene D5 in pea, an increasing number of scholars have conducted research on the entire MAPK gene family in plants such as *Arabidopsis thaliana* (MAPK Group, 2002), wheat (Ergen et al., 2009), and lettuce (Wang et al., 2022). In terms of evolutionary relationships, MAPK proteins in plants are divided into four subgroups: A, B, C, and D. Among them, A, B, and C belong to the TEY type, whereas the TDY type contains only the D subgroup. MAPK proteins containing TDY motifs are found only in plants. The common docking domain (CD) conserved domain exists in some MAPK proteins with the conserved motif (LH) DXXDE (P) X, which is usually highly conserved in groups A and B. The CD domain of group C has been modified, whereas group D does not contain this domain (Shang et al., 2023).

The MAPK cascade pathway is involved in almost all life processes of plants and plays a key role in plant growth and development, especially under biological and abiotic stresses (Shao et al., 2020). Among them, the functions of the *A. thaliana* MAPK family members *AtMPK3*, *AtMPK4*, and *AtMPK6* have been widely studied. *AtMPK4* negatively regulates plant disease resistance and osmotic stress tolerance. Together with *AtMPK6*, *AtMPK3* participates in regulating auxin polar transport and promoting ethylene synthesis. In addition to *A. thaliana*, 16, 18, 12, and 11 MAPK genes have been identified in rice (Hamel et al., 2006), kiwifruit (Wang et al., 2018), grape (Zhan et al., 2017), and cotton (Zhang et al., 2016), respectively, where they have been found to contain a certain number of salt tolerance genes and participate in the regulation of MAPK cascade pathways upstream and downstream.

The mainstream strategy of using microbial agents to enhance the plant response to abiotic stress has become a major trend in resistance biology (Liu et al., 2010; Saberi et al., 2021). *Bacillus subtilis* (Bs), a gram-positive bacterium capable of surviving in extremely hot environments, has drawn widespread attention because of its strong adaptability and potential role in resisting abiotic stress, as discovered in microbiological engineering (Xiao et al., 2024). This spore-forming bacterium can withstand harsh environments and promote plant growth by forming biofilms in the rhizosphere or establishing symbiotic relationships with roots. This capability enhances the production of stress proteins, antioxidants, and growth hormones, facilitating plant growth. It aids in maintaining cellular osmotic regulation and the water balance under salt stress conditions. Recently, researchers have discovered correlations between the transcriptomes of cotton and *B. subtilis*, thereby enhancing biotic stress tolerance. The mechanism by which Bs enhances plant salt tolerance by excreting Na^+^ ions has been revealed and demonstrated in important crop species, such as rice and clover. Our previous experiments validated the effectiveness of enhancing licorice salt tolerance via the inoculation of Bs, and multiple omics analyses revealed the key metabolic pathways and signaling pathways involved (Chardin et al., 2017). The MAPK pathway, which is crucial for regulating abiotic stress responses by modulating cell division, was notably enriched in the KEGG pathway database. Previous studies have elucidated only the basic signaling pathways and key differentially expressed genes of MAPK (Xiao et al., 2024). Through bioinformatics analysis, we aimed to gain deeper insights into the function and diversity of the *GuMAPK* family in response to salt stress. In this study, we identified MAPK gene family members in the *G. uralensis* genome based on bioinformatics, analyzed their chromosomal positions, evolutionary relationships, conserved motifs and domains, *cis*-acting elements, and phosphorylation sites, and studied the expression levels of *GuMAPK* genes under salt stress and after *B*. *subtilis* inoculation through transcriptome and protein interaction analysis. These findings provide further evidence for the in-depth exploration of the mechanism by which *B. subtilis* enhances *G. uralensis* salt tolerance.

## 2 Materials and methods

### 2.1 Plant materials

The *Glycyrrhiza uralensis* seeds used were identified and preserved in our laboratory. Full and intact *G. uralensis* seeds were first soaked in 75% ethanol for 30 s and then in a 3% sodium hypochlorite solution for 15 min on an aseptic operating table. They were subsequently rinsed with sterile water at least three times until neutral and then placed in a 4°C refrigerator for 3 days of stratification before being transferred to a constant 28°C incubator for a 7-day dark treatment. Once most seeds displayed a whitish appearance, they were planted in pots and cultivated for 50 days in a well-lit room.

The salt stress treatment commenced when the *G. uralensis* plants had grown 8–10 true leaves. We established two salt stress concentrations: 100 mmol/L (NCL) and 300 mmol/L (NCH) NaCl + CaCl_2_, while the control group (NC0) received no NaCl + CaCl_2_. In addition, *B*. *subtilis* was inoculated as the *B. subtilis* group, which was divided into Bs0, BsL, and BsH. The seedlings were inoculated with *B. subtilis* spores by spraying them once every 15 days on the soil surface (with an effective viable count ≥50 billion CFU/g). All of the solutions were prepared with 1/2 Hoagland nutrient solution. In total, four waterings were conducted, with a 6-day interval between each. Root tissues from seedlings at the same growth stage that exhibited robust growth were promptly collected, flash-frozen in liquid nitrogen, and stored at −80°C in a freezer for transcriptome sequencing. The tobacco cultivation method used for the subcellular localization experiments was previously described by Zhang et al., (2023).

### 2.2 Genome-wide identification of *GuMAPK* family members in *Glycyrrhiza uralensis*

The analysis of the MAPK gene family in *G. uralensis* was conducted by first downloading the full genome data from the JGI database (https://phytozome.jgi.doe.gov/pz/portal.html). The serial/threshold protein kinase domain (PF00069) Hidden Markov Model file was obtained from the PFAM database (http://pfam.xfam.org/). The Hmmsearch tool was then employed to align the *G. uralensis* genome protein data with the MAPK Hidden Markov Model, selecting protein sequences with an E value ≤ e^−10^ as candidate MAPK family members. The candidate protein sequences were further validated for conserved domains, using the PFAM website to remove those lacking MAPK domains and repetitive sequences, ultimately retaining 21 MAPK family genes. The physical and chemical characteristics of the MAPK proteins, such as the number of amino acids, molecular weight, isoelectric point (pI), and hydrophobicity, were predicted using the online tool ExPASy (http://web.expasy.org/protparam/). The subcellular localization of *GuMAPKs* was predicted using the Busca website (http://busca.bio-comp.unibo.it/). Furthermore, protein secondary structure analysis was performed using the SOPMA website (https://npsa-prabi.ibcp.fr/) (Savojardo et al., 2018).

### 2.3 Sequence alignment and phylogenetic analysis

MEGA 11.0 software was used to construct a phylogenetic tree of the MAPK proteins from *G. uralensis* and *A. thaliana* using the ClustalW algorithm. The tree-building method should employ the maximum likelihood approach, with all other parameters set to default values. The phylogenetic tree was exported and further enhanced in appearance and color scheme using the Evolview online tool (https://evolgenius.info//evolview-v2/). Additionally, Jalview software was used to refine multiple sequences and create a sequence logo for each MAPK domain using the WebLogo 3 online tool.

### 2.4 Gene structure, conservation domain identification, and 3D structure prediction

MEME (https://meme-suite.org/meme/) was used for online analysis to identify conserved motifs in the *GuMAPK* proteins (Timothy et al., 2015). Simultaneously, the conserved domains of *GuMAPK* proteins were predicted, limiting the number of motifs to “10” while keeping the other parameters at the default values. Structural information for the *GuMAPK* genes was extracted from the downloaded *G. uralensis* genome database. GSDS (http://gsds.gao-lab.org/) was used to generate gene structure diagrams, which depict the distributions of exons, introns, and untranslated regions (UTRs) (Hu et al., 2015). The three-dimensional structure of *GuMAPK* proteins was predicted via SWISS-MODEL (https://swissmodel.expasy.org/) via homology modeling. Templates with sequence identities greater than 30% were selected, and protein models were evaluated via SAVESv6.0 (https://saves.mbi.ucla.edu/). Templates validated by three or more evaluations were chosen as the final templates. The protein structures were visualized with the 3D protein structure visualization software VMD (http://www.ks.uiuc.edu/Research/vmd) (Humphrey et al., 1996).

### 2.5 Chromosomal localization and collinearity analysis

The chromosomal location data of MAPK genes from the *G. uralensis* genome files were collected and organized. TBtools was used for analysis and visualization of gene location distributions (Chen et al., 2020). Basic Circos analysis was conducted to investigate gene duplication events among *GuMAPK* members using the default parameters. The genome data of *A. thaliana*, tomato (*Solanum lycopersicum*), and cucumber (*Cucumis sativus*) were downloaded from the Ensembl website. Interspecies collinearity analysis was performed via the plug-in tools Advanced Circos and One Step MCScanX in TBtools.

### 2.6 Prediction of *cis*-acting elements and phosphorylation sites

To identify potential *cis*-acting elements, the promoter region sequences of *GuMAPK* genes, which span 2,000 base pairs upstream of the transcription start site, were extracted from the genome data. *Cis*-acting elements in the upstream region were predicted via the Plant CARE website (http://bioinformatics.psb.ugent.be/webtools/plantcare/html/), and these elements were annotated. Focus should be placed on plotting the positional distributions of the elements related to hormones, the stress response, and growth and development. Phosphorylation analysis of the *G. uralensis* MAPK gene family was conducted via NetPhos (https://services.healthtech.dtu.dk/service.php?NetPhos-3.1), which revealed the distribution of protein kinase phosphorylation sites within *GuMAPKs*.

### 2.7 RNA extraction, transcriptome and qRT-PCR analysis under salt stress with *Bacillus subtilis* inoculation

For each treatment, 0.5 g of root tissue was obtained from seedlings exposed to salt concentrations of 0, 100 or 300 mmol/L. These samples were subsequently frozen in liquid nitrogen. RNA extraction from the root tissue samples of *G. uralensis* seedlings (inclusive of three biological replicates) was conducted using TRIzol^®^ Reagent (Invitrogen, United States). The extracted RNA was purified with AMPure XP beads (FuSheng, Shanghai, China) and subsequently analyzed utilizing the Marjorie BioPharma Technology Co. HiSeq 4000 Illumina sequencing platform in China. Each group’s sequencing results consisted of three independent biological replicates. The average values were used to plot the expression levels of each gene across different groups using the Bioinformatics platform.

The RNA acquired was subjected to reverse transcription, resulting in the generation of complementary DNA (cDNA). Subsequently, differentially expressed gene-specific primers were employed for quantitative polymerase chain reaction (qPCR) utilizing the double-stranded chimeric fluorescent dye method, specifically employing SYBR Green I. An internal reference gene, β-actin, was utilized, and the average of three biological replicates was calculated. Gene expression was determined using the 2^−ΔΔCt^ method.

### 2.8 Cloning and subcellular localization of *GuMAPK16-2*

Glyur001680s00029814 was isolated from *G. uralensis* using *GuMAPK16-2*-F/R primers. The coding sequence (CDS) of *GuMAPK16-2*, lacking a stop codon, was inserted into the TA cloning vector before being subcloned and inserted into the pBWA(V)HS:GFP vector. Following construction, the vectors were introduced into *Agrobacterium tumefaciens* strain GV3101. The resulting fusion protein, pBWA(V)HS: GFP: *GuMAPK16-2*, along with the control pBWA(V)HS: GFP, was transiently expressed in tobacco leaves. Following infection, the tobacco plants were cultivated in a light-protected environment at 24°C for 2 days. Fluorescence visualization was subsequently conducted using laser confocal microscopy (Zeiss, Germany).

## 3 Results

### 3.1 Identification, chromosomal localization and physicochemical property analysis of *GuMAPKs*

**FIGURE 1:**
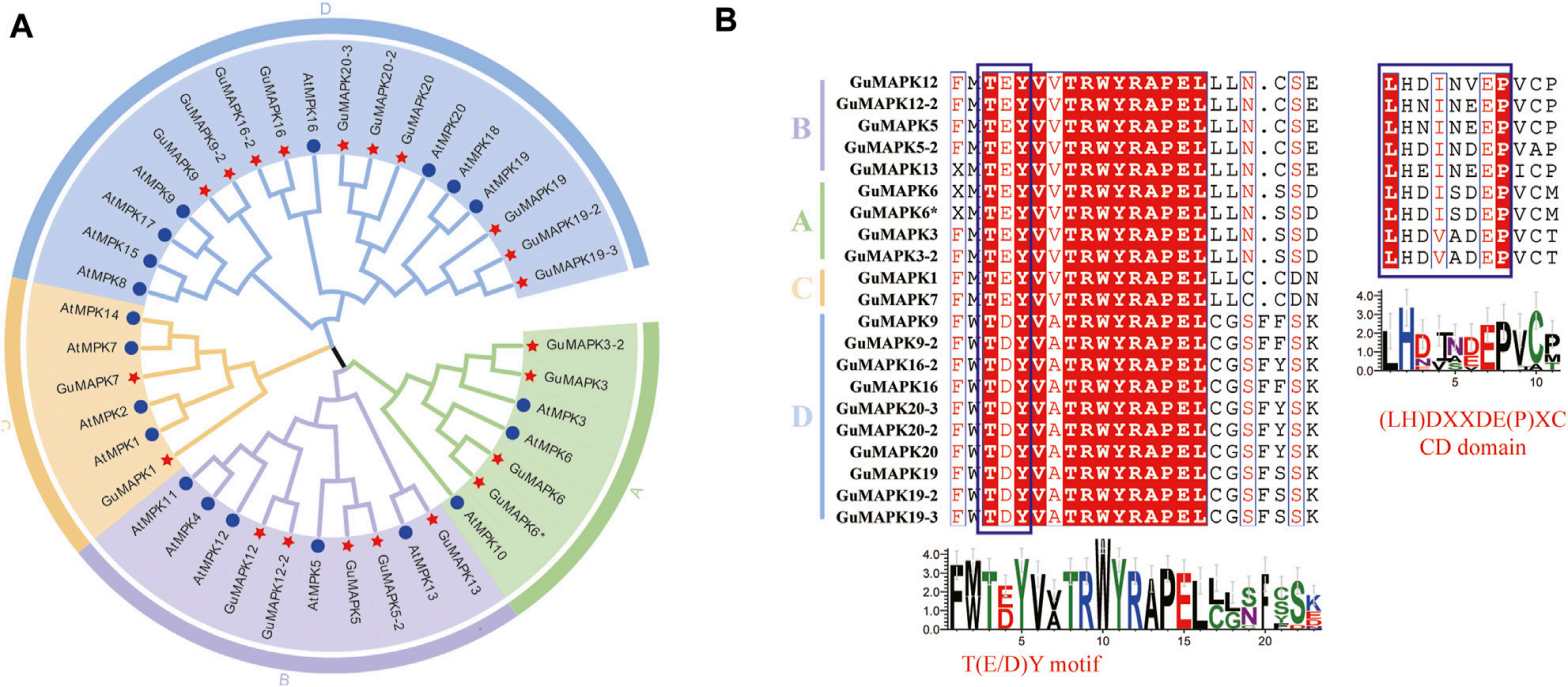
Phylogenetic relationships and multiple sequence alignment analysis of *GuMAPKs*. **(A)** Full-length MAPK sequences from *Arabidopsis thaliana* and *Glycyrrhiza uralensis* were used to construct a rootless phylogenetic tree. Different colors represent different branches, A-D indicate the different groups of MAPKs. **(B)** Multiple sequences alignment of the conservative domain from *GuMAPK* proteins. Dark red highlighted residues are identical, while red words high-lighted residues are similar in all proteins. Blue boxes indicate the TEY/TDY motifs and the CD domain.

Through comprehensive bioinformatic analyses, including homology comparisons and domain screening, 21 reliable members of the MAPK gene family were identified in the *G. uralensis* genome. On the basis of an in-depth analysis of evolutionary relationships between *GuMAPK* and *AtMAPK*, a unified nomenclature system was established, leading to the systematic renaming of the identified genes accordingly (Figure 1A). Notably, *GuMAPK6* and GuMAPK6* were found to be identical genes. The distribution of members of the *G. uralensis* MAPK gene family on scaffolds, along with their physicochemical properties, were analyzed (Supplementary Table S1). The results revealed that the molecular weights of the *GuMAPKs* ranged from 42,620.78 to 72,510.22 Da, with isoelectric points (pIs) varying between 5.20 and 9.22. The length of the amino acid sequences ranged from 369 to 638 aa. Stability prediction of their proteins revealed instability coefficients ranging from 34.32 to 45.55, with 11 *GuMAPKs* identified as unstable proteins. The hydrophobicity prediction indicated that the grand average hydropathy (GRAVY) score range was −0.619 to −0.113. According to the subcellular localization prediction, except for one *GuMAPK* (*GuMAPK12-2*) located in the chloroplast, six *GuMAPKs* (*GuMAPK1*, *GuMAPK3*, *GuMAPK3-2*, *GuMAPK7*, *GuMAPK12*, *GuMAPK13*) were in the cytoplasm, whereas the remaining 14 *GuMAPKs* were in the nucleus.

### 3.2 Systematic evolutionary analysis and Multiple Sequence Alignment Analysis of *GuMAPKs*

To validate the homologous relationship between the *GuMAPKs* and *AtMAPKs* gene families, a phylogenetic tree was constructed using the protein sequences of 21 *GuMAPKs* and 20 *AtMAPKs* (Figure 1A). *GuMAPK* proteins were categorized into four subgroups on the basis of the classification method and clustering characteristics of *A. thaliana*: A (4 members), B (5 members), C (2 members), and D (10 members). Subgroup D presented the highest membership, whereas subgroup C presented the lowest membership. Subgroups A and B clustered together on the same branch. Multiple sequence alignment analysis revealed that all GuMAPKs contained a conserved tripeptide motif, TXY, with subgroups A, B, and C featuring TEY and subgroup D featuring TDY. Furthermore, members of subgroups A and B also shared a conserved CD domain (Figure 1B).

### 3.3 Gene structure, conserved motif and conserved domain analysis of *GuMAPKs*

**FIGURE 2:**
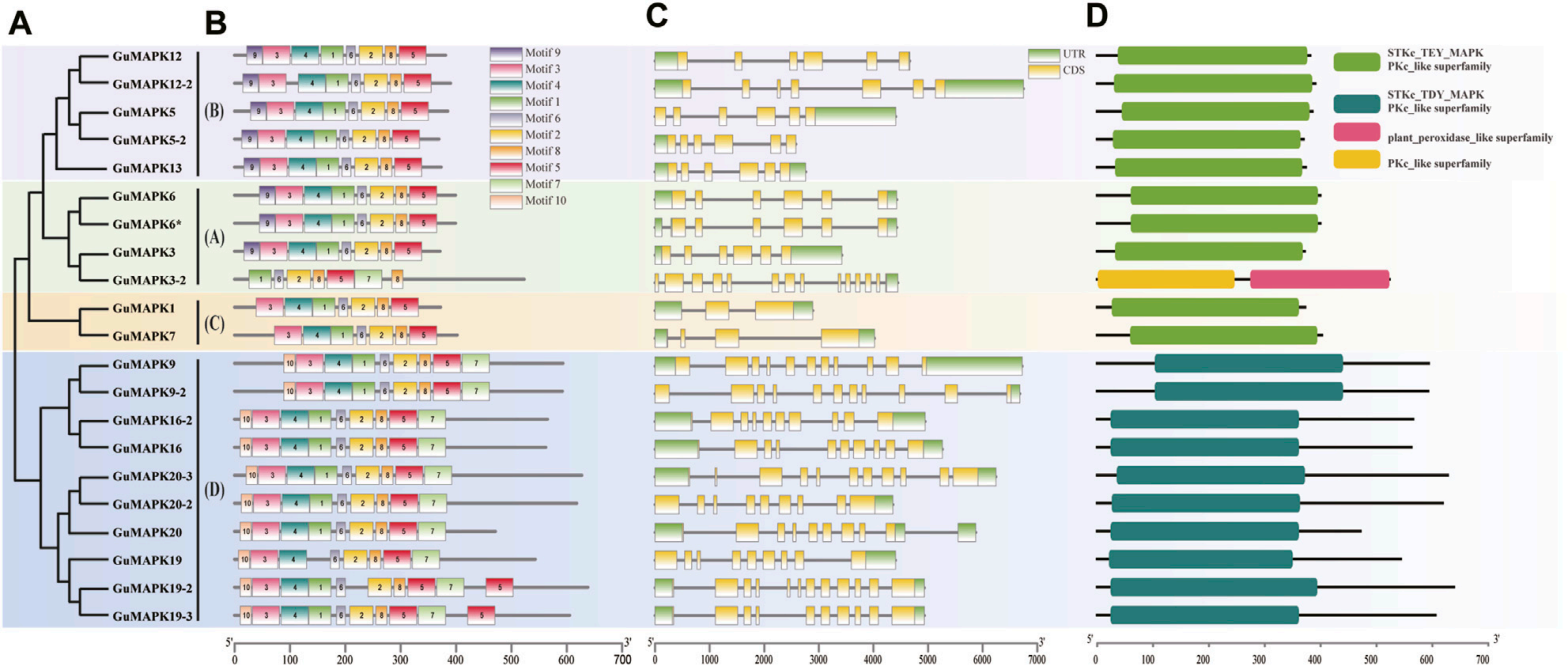
Analysis of *GuMAPK* genes conserved motifs and gene structure. **(A)** *GuMAPK* protein phylogenetic tree, different colors were used to distinguish groups. **(B)** The distribution of onserved motifs in *GuMAPK* proteins. Each motif is represented by a colored box. **(C)** Gene structure analysis of *GuMAPKs*. **(D)** Conservative domain analysis of GuMAPKs.

In the prediction of motifs for licorice *GuMAPK* proteins, 10 motif sequences were identified. Among them, motif 6 represents the highly conserved TRWYRAPEL domain found in MAPKs, which is present in all *GuMAPKs*. Motif 9 corresponds to the conserved CD domain present in all members of the A and B subgroups except for *GuMAPK3-2*. All members of the *GuMAPK* family contain motifs 1, 6, 2, 8, and 5, which are consistently located, suggesting their pivotal role in the evolutionary history of the *GuMAPK* gene family. Some motifs are specific to certain subgroups and highly conserved among members within the same subgroup. Additionally, motifs 7 and 10 are exclusively present in the D subgroup, with the exception of *GuMAPK3-2*, which contains motif 7 (Figures 2A, B).

Gene structure analysis revealed that members of the *GuMAPK* gene family contain 2 to 14 exons and 2 to 13 introns (Figure 2C). Except for *GuMAPK12-2* (B) and *3-2* (A), members of the A and B subgroups typically have six exons and five introns. In contrast, members of the C subgroup exhibit simpler gene structures; *GuMAPK1* has two exons and two introns, whereas *GuMAPK7* has three exons and three introns, showing significant differences compared with the other subgroups. This implies that members belonging to the same subgroup exhibit comparable genetic architectures. *GuMAPK3-2* has the greatest number of exons and introns, with 14 exons and 13 introns. Members of the D subgroup have more complex gene structures, ranging from 9 to 12 exons and 8 to 11 introns. Moreover, most genes within the same subgroup, such as *GuMAPK5* and *13* in the B subgroup, and *GuMAPK20* and *19* in the D subgroup, exhibit similar exon‒intron structures, indicating a conserved exon‒intron distribution pattern among these GuMAPKs. Overall, the gene structure characteristics of *GuMAPK* family members demonstrate both intergroup diversity and intragroup conservation.

The analysis of conserved domains revealed that all *GuMAPK* proteins possess the PKc_like superfamily domain. Specifically, members of the A, B, and C subgroups typically contain the STKc_TEY_MAPK domain, whereas members of the D subgroup usually harbor the STKc_TDY_MAPK domain. Additionally, *GuMAPK3-2* also features a plant_peroxidase_like superfamily domain, distinguishing it from other members (Figure 2D).

### 3.4 Collinearity analysis of *GuMAPKs*

**FIGURE 3:**
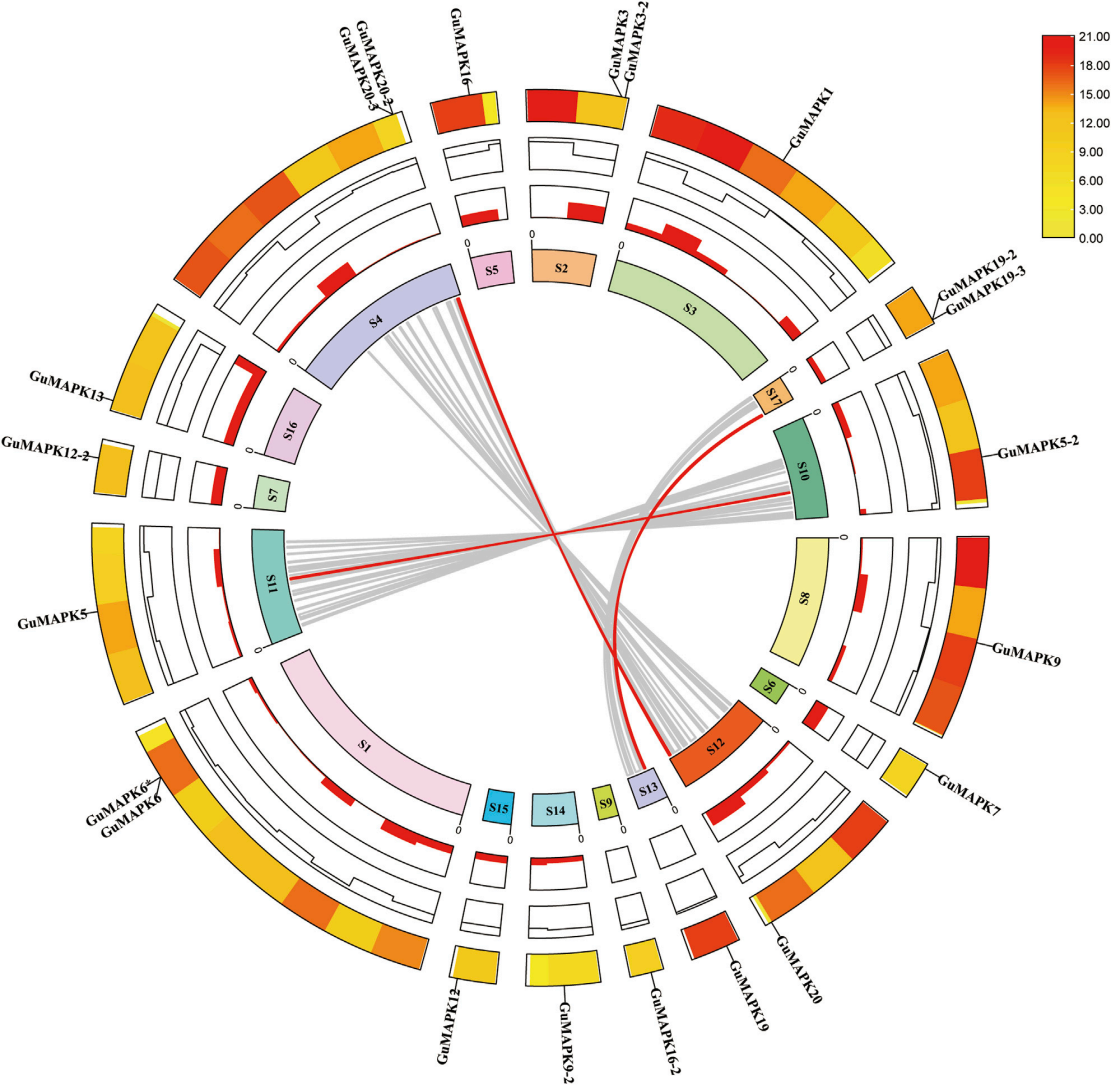
Collinearity analysis of the *GuMAPK* gene family. The gray lines in the background represent homologous pairs of all *Glycyrrhiza uralensis* genes in these 17 chromosome scaffolds, and the red line represents the homolinear pair of *GuMAPKs*. The information represented by each circle in the figure is chromosome scaffolds name, GC radio, gene density heat map and *GuMAPKs* chromosome location annotation from inside to outside. Yellow to red on the heat map indicates low to high gene density.

Analysis of the chromosomal location of the *GuMAPK* gene family members revealed that the 21 *GuMAPKs* are unevenly distributed across 17 different chromosome scaffolds. Among them, *GuMAPK6* and GuMAPK6* are located on the same chromosome scaffold (Supplementary Figure S1). Further analysis of collinearity helps elucidate the potential evolutionary mechanisms of the *G. uralensis* gene family (Figure 3). Therefore, an analysis of gene duplication events involving *GuMAPK* genes on chromosome scaffolds was conducted. The results indicate that out of 97 pairs of homologous genes distributed across these 17 chromosome scaffolds, six scaffolds—Scaffold00074, Scaffold01036, Scaffold00100, Scaffold00268, Scaffold00029, and Scaffold00028—contained homologous gene pairs. Among all homologous gene pairs, three pairs belong to *GuMAPK*, and all of them represent fragment duplications. Thus, fragment duplication events appear to be the main driving force behind *GuMAPK* evolution.

**FIGURE 4:**
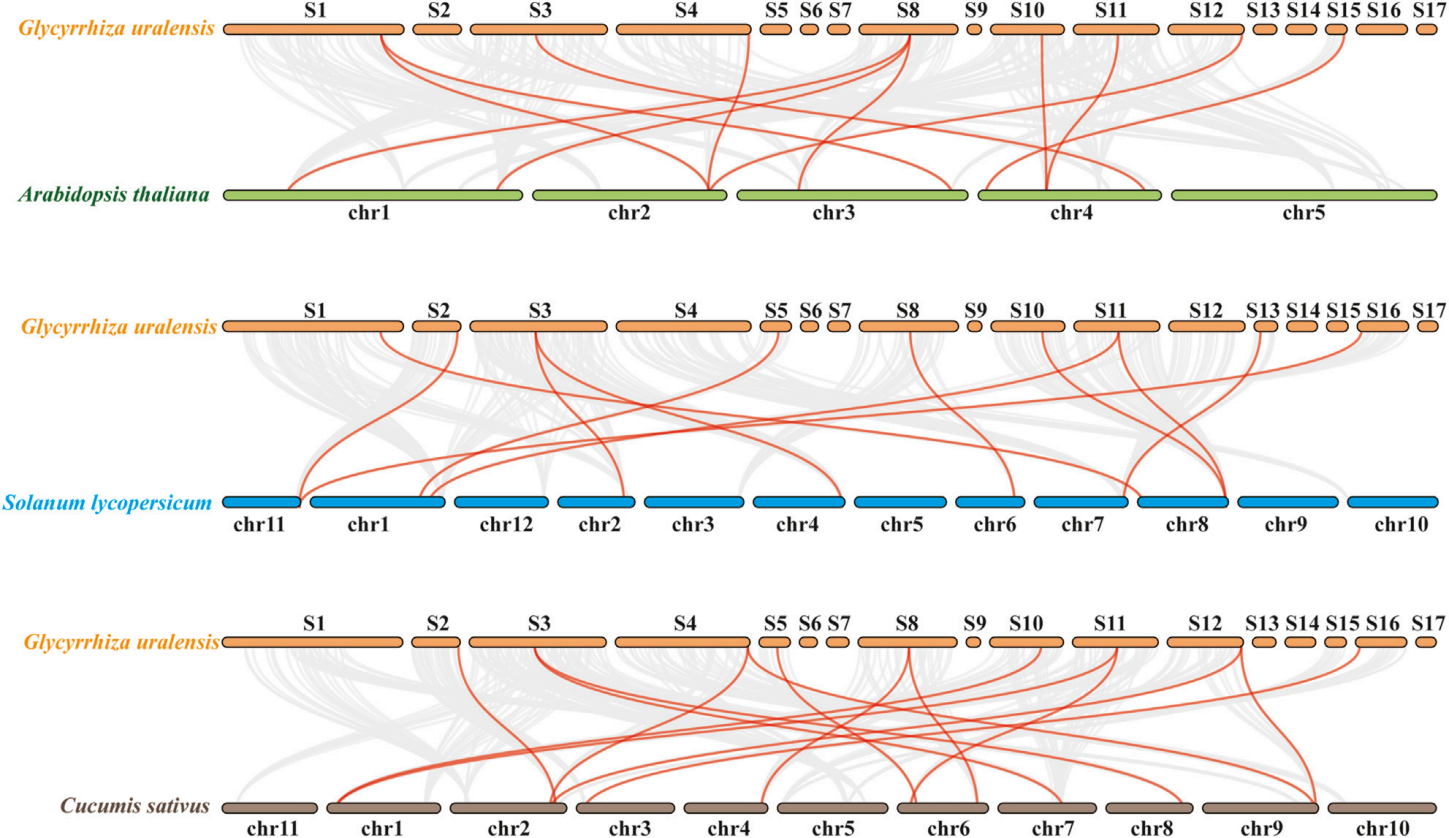
Collinearity analysis of *Glycyrrhiza uralensis* with *Arabidopsis thaliana*, *Solanum lycopersicum* and *Cucumis sativus*. The gray line in the back-ground represents all collinear pairs of *Glycyrrhiza uralensis* with *Arabidopsis thaliana*, *Solanum lycopersicum* and *Cucumis sativus* at the genomic level. The red line represents the collinear pairs belonging to MAPK family members from those four species.

Additionally, at the genomic level, collinearity analysis was performed between *G. uralensis* and *A. thaliana*, tomato, and cucumber (Figure 4). The results revealed collinearity between *G. uralensis* and *A. thaliana* for 319 pairs, *G. uralensis* and tomato for 312 pairs, and *G. uralensis* and cucumber for 356 pairs, with 11, 11, and 14 pairs, respectively, belonging to the *GuMAPK* gene family. Notably, the *GuMAPK1, 5, 5-2,* and *9* genes exhibited collinearity with genes in *A. thaliana*, tomato, and cucumber, suggesting their significant role that requires them to be conserved in the evolutionary process.

### 3.5 *Cis*-acting element analysis and phosphorylation site analysis of *GuMAPKs*

**FIGURE 5:**
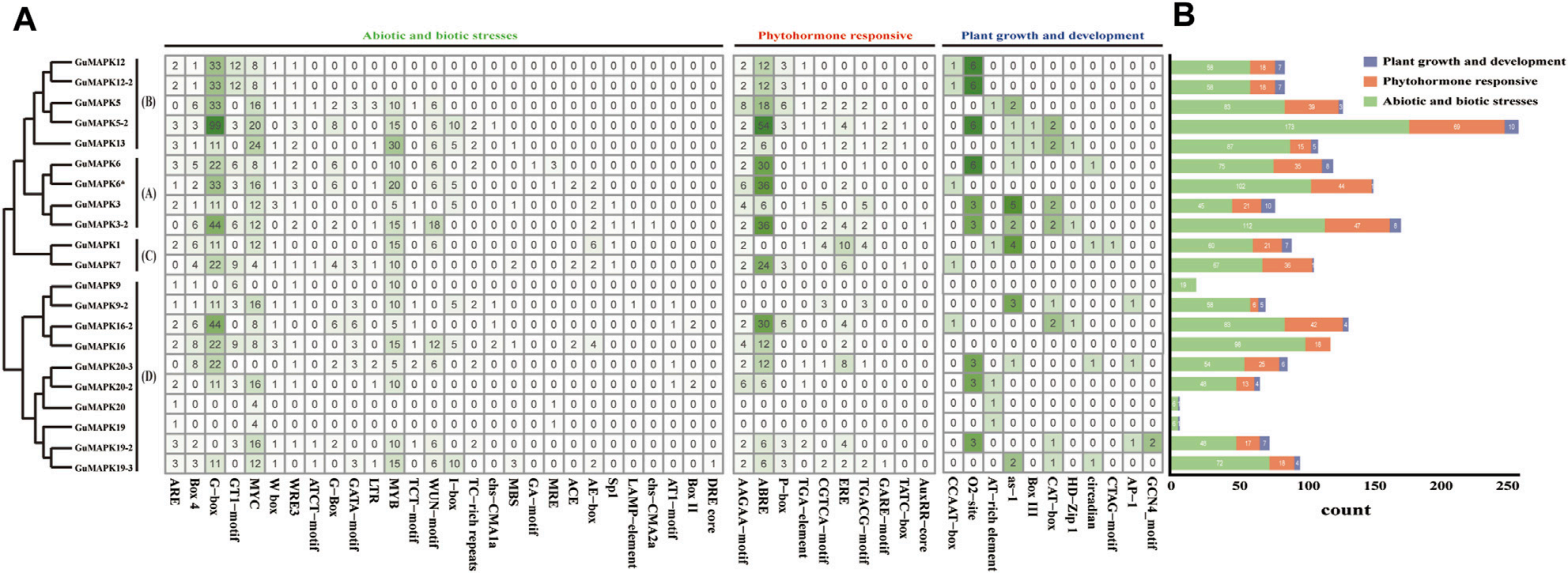
Distribution of the cis-acting elements in the promoters of *GuMAPKs*. **(A)** Quantitative heatmap of *cis*-acting elements in *GuMAPKs*. **(B)** Statistics on the number of cis-acting elements classified in *GuMAPKs*.

Through analysis of the 2,000 bp upstream sequences of *GuMAPK* promoters, a total of 51 *cis*-acting elements were identified (Figure 5A). In addition to the core promoter elements TATA box and enhancer element CAAT box, the remaining 49 elements were classified into three categories: plant growth and development (11), plant hormone response (10), and biotic and abiotic stress (28). In addition to *GuMAPK9*, which contains only biotic and abiotic stress elements, and *GuMAPK19* and *20*, which lack plant hormone response elements, members of each category are distributed among all *GuMAPKs.* Among them, the elements related to biotic and abiotic stress are the most abundant (1,412), while those related to plant growth and development are the least common (99). Heatmap analysis of the *cis*-acting elements in the three categories revealed that among the 2013 elements (Figure 5B), the most frequently occurring elements were G-boxes (99 occurrences) and ABREs (54 occurrences). The B-group members *GuMAPK12* and *12-2* and the D-group members *GuMAPK19* and *20* showed consistency in the classification and quantity of *cis*-acting elements, suggesting that they might have similar functions. Although the coding sequences (CDSs) of *GuMAPK6* and 6* are identical, differences in *cis*-regulatory elements may result in distinct expression patterns, suggesting different functional scenarios for these two genes.

**FIGURE 6:**
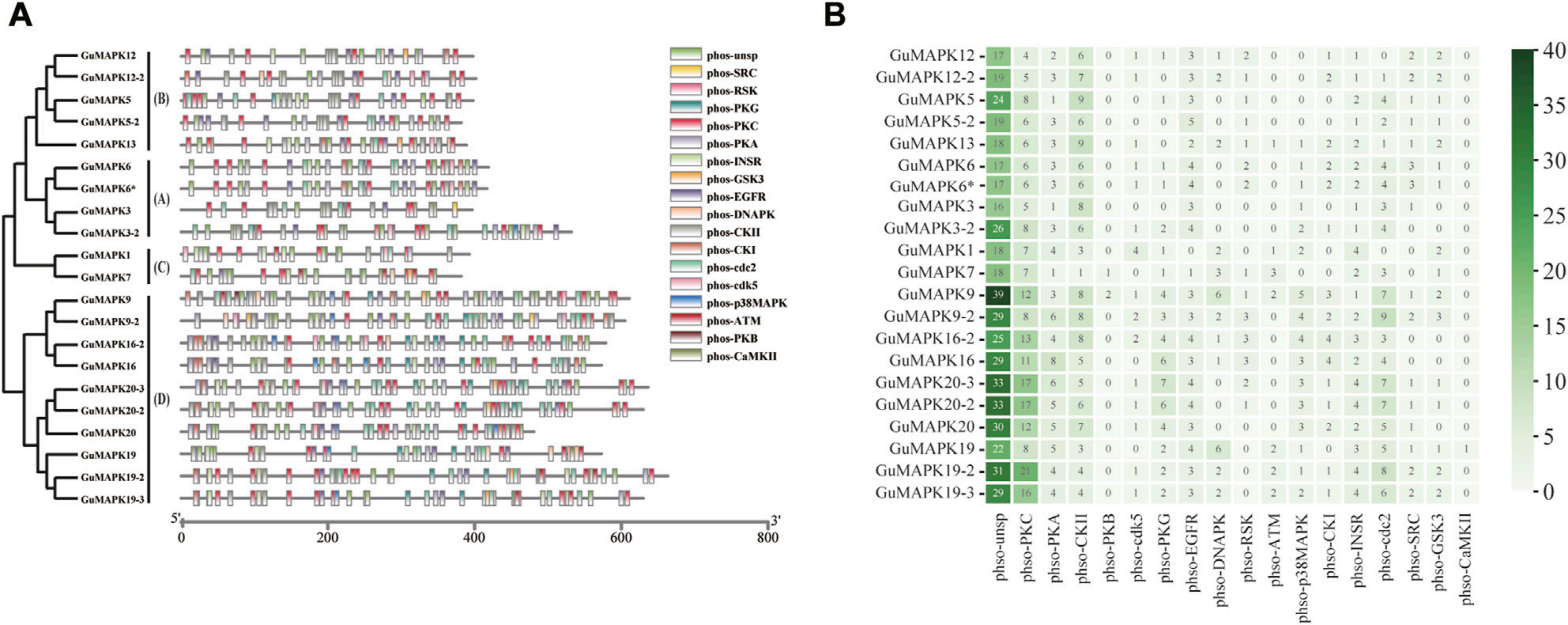
Prediction of phosphorylation sites of *GuMAPKs*. **(A)** Distribution of phosphorylation sites of protein kinases in *GuMAPKs*. **(B)** Quantitative heatmap of protein kinases at phosphorylation sites. Green from light to deep indicates the number of phosphorylation sites from less to more.

Protein kinases are typically activated through phosphorylation, highlighting the importance of investigating phosphorylation sites to understand their functional mechanisms. In this study, phosphorylation prediction was conducted on the *GuMAPK* gene family. The results revealed a total of 1,370 phosphorylation sites among *GuMAPKs*, which were widely distributed across all *GuMAPK* sequences (Figure 6A). Among these, the most abundant were nonspecific phosphorylation sites (Phos-unsp), totaling 509; followed by specific sites for phosphorylation by protein kinase C (Phos-PKC), with 203 sites; and sites for casein kinase II (phso-CKII), totaling 125. Quantitative heatmap analysis of all of the phosphorylation sites revealed that *GuMAPK9* had the greatest number of total phosphorylation sites (100) and specific phosphorylation sites (61), which may be related to its amino acid sequence length (Figure 6B). However, the quantity of phosphorylation sites does not always correspond with the length of the amino acid sequence. Despite *GuMAPK13* having a shorter amino acid sequence than *GuMAPK3*, *GuMAPK13* presented a richer distribution of phosphorylation sites. *GuMAPK6* and 6* share identical distributions of phosphorylation sites, likely due to their identical sequences. Additionally, only one site for phosphorylation by calcium/calmodulin-dependent protein kinase II (phso-CaMKII) was predicted in *GuMAPK19*, possibly indicating a distinct functionality.

### 3.6 Prediction of the secondary structure and three-dimensional structures of *GuMAPK* proteins

**FIGURE 7:**
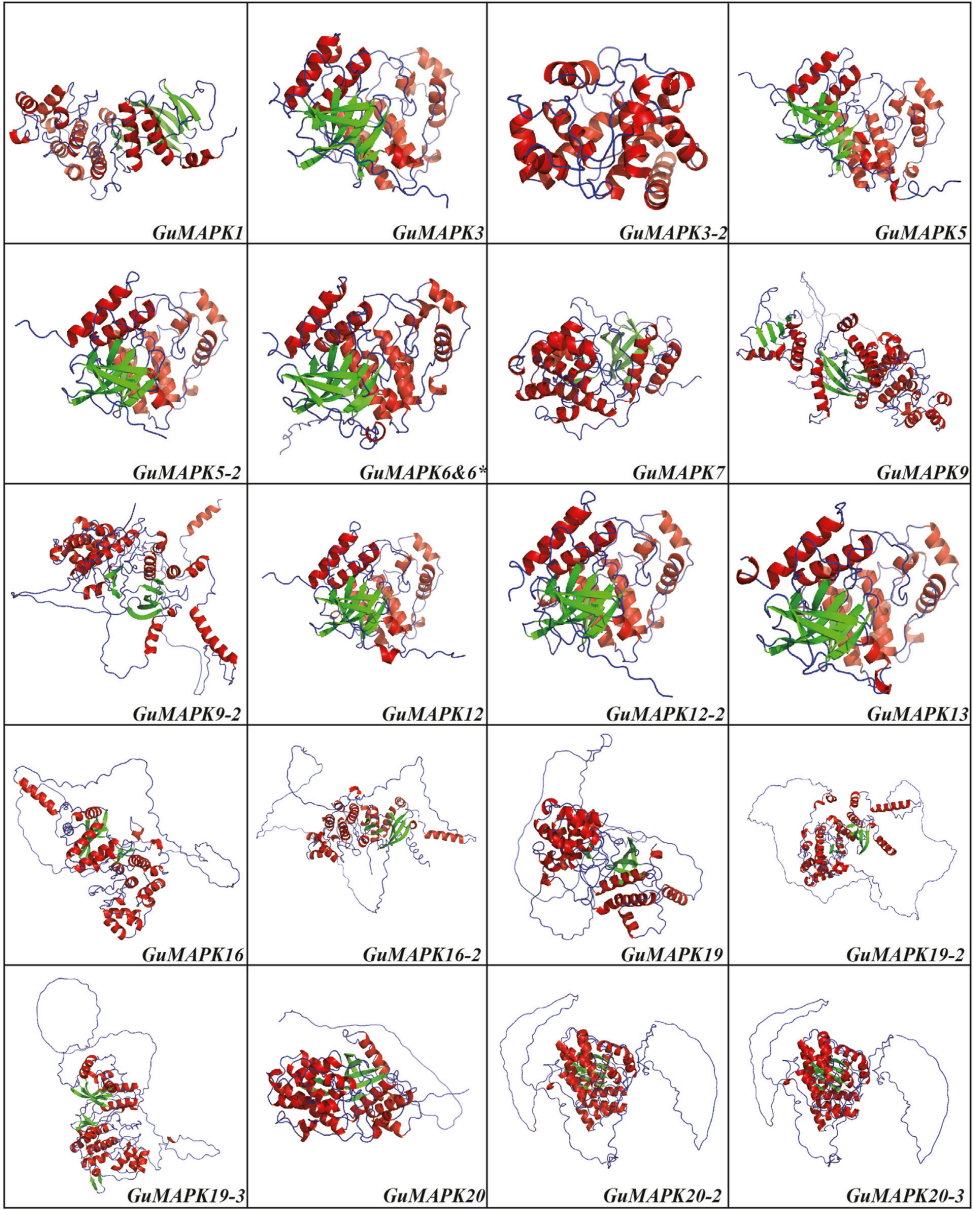
3D structure prediction for *GuMAPKs*. The alpha helix is shown in red, the beta fold is shown in green, and the irregular curl is shown in blue.

The diverse functionalities of proteins are closely correlated with their complex structures. To further explore this relationship, the secondary and tertiary structures of the 21 *GuMAPK* proteins were predicted. The secondary structure prediction revealed that the peptide chains of all *GuMAPK* family members predominantly consisted of α-helices, β-turns, extended strands, and irregular coils. Among these, β-turns were the least abundant, constituting less than 9% of the total structure in all 21 *GuMAPKs*. In the A, B, and C groups, α-helices and irregular coils accounted for more than 40% and 30% of the structure, respectively, with α-helices being the primary conformation, accounting for more than irregular coils. Conversely, in the D group, irregular coils were the predominant conformation, comprising more than 40% of the structure (Supplementary Table S2). Using homology modeling, the three-dimensional structures of the 21 *GuMAPK* proteins were predicted (Figure 7). Except for the consistent structures of *GuMAPK6* and 6*, the three-dimensional structures of the other 20 genes were accurately evaluated using online software. The results revealed that all family members, except *GuMAPK3-2*, contained β-folds in addition to α-helices and irregular coils. Notably, the D group members presented a greater proportion of irregular coils, which was consistent with the secondary structure predictions.

### 3.7 Expression analysis under salt stress with *Bacillus subtilis* inoculation and network interaction analysis

**FIGURE 8:**
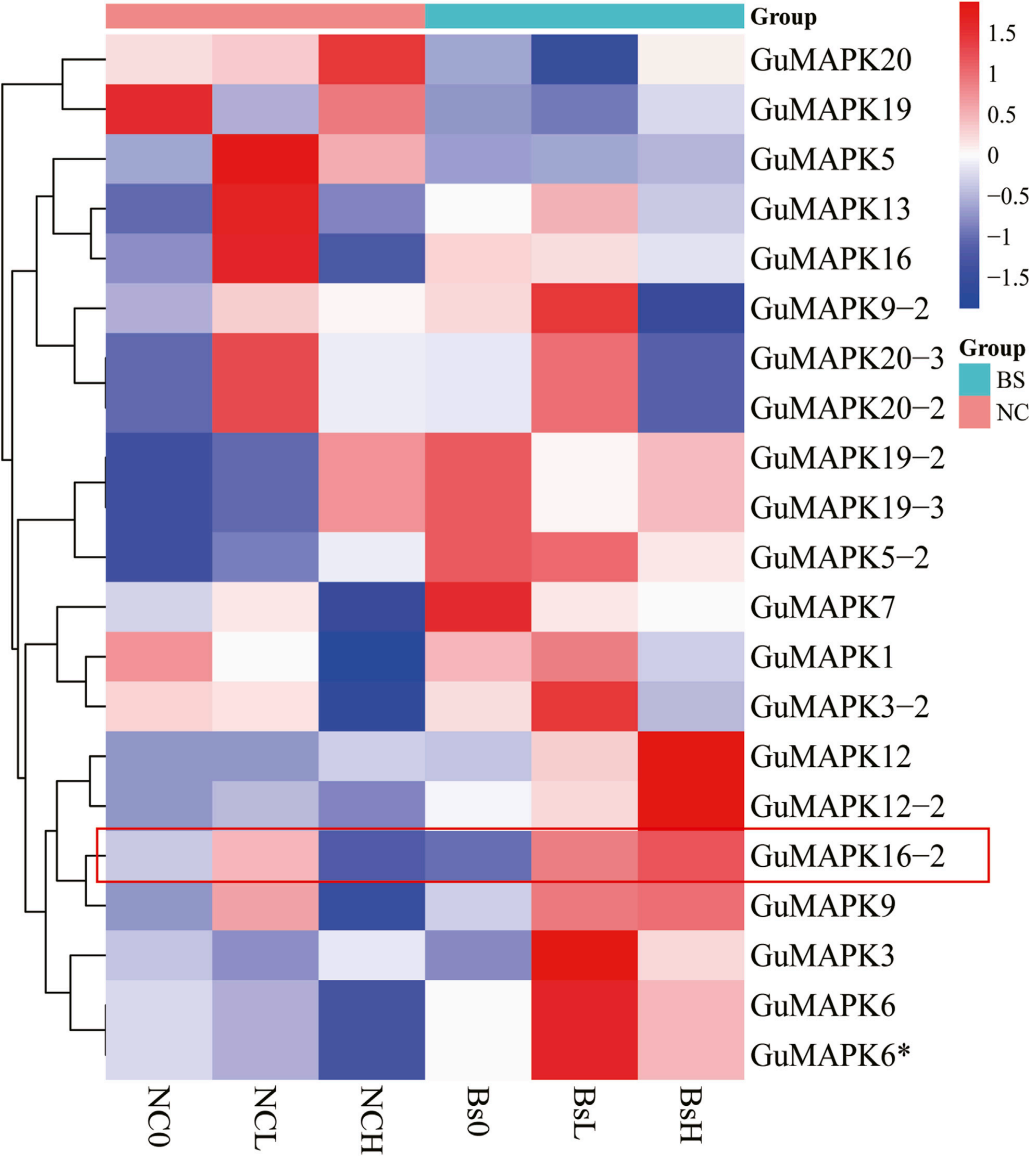
Transcriptional abundance of *GuMAPK* genes in response to salt stress by inoculating *Bacillus subtilis*. Colored bars indicate relative abundance in Z-Score, red indicates high level expression, and dark blue indicates low expression. NC group means that salt solution with different concentration is used to simulate salt stress environment, and Bs group means that *Bacillus subtilis* is inoculated on the basis of salt stress in NC group.

**FIGURE 9:**
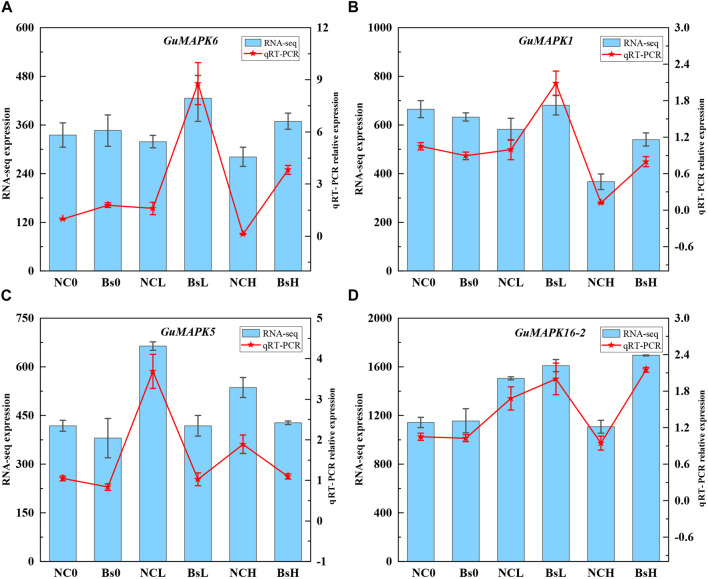
Plot of qRT-PCR analysis of some differentially expressed genes. **(A)** GuMAPK6. **(B)** GuMAPK1. **(C)** GuMAPK5. **(D)** GuMAPK16-2. The blue bars represents the count value of the genes at the transcriptome level,which corresponds to the left Y-axis, and the red fold represents the relative expression of the genes, which corresponds to the right Y-axis. Each group has three independent biological replicates.

Transcriptomic data were extracted and subjected to cluster analysis to comprehensively investigate the expression profiles of the 21 members of the MAPK gene family in *G. uralensis* under salt stress induced by inoculation with *B*. *subtilis*. The results revealed that under low (100 mmol/L) and high (300 mmol/L) concentrations of NaCl + CaCl_2_ solution, 18 genes were induced under salt stress (including *GuMAPK6* and 6*, *19-2* and *19-3*, *20-2* and *20-3*, which represent the same genes). Particularly noteworthy is the significant downregulation of *GuMAPK16-2* under 300 mmol/L salt stress (Nc0 vs. NcH, *P* = 0.038), whereas its expression was significantly upregulated compared with that under *B*. *subtilis* inoculation (NcH vs. BsH, *P* = 0.030) (Figure 8). Furthermore, under low salt concentrations, the expression levels of the genes *GuMAPK3*, *GuMAPK6*, and GuMAPK6* increased by 3.05-fold, 1.33-fold, and 1.33-fold, respectively, following inoculation with *B. subtilis*. These genes not only cluster together in their expression patterns but also belong to subgroup A, implying that subgroup A genes may effectively alleviate salt stress when inoculated with *B. subtilis* under mild salt stress conditions. To verify the reliability of the transcriptome data, *GuMAPK1, 5, 6,* and *16-2* were selected for qRT‒PCR validation (Supplementary Table S3), and the results were consistent with the trends observed in the transcriptome analysis (Figure 9).

To examine *GuMAPK* protein function, an interaction network diagram was devised utilizing preliminary research findings and homologous protein families found in *A. thaliana* (Supplementary Figure S2). By leveraging the well-studied functions of *AtMAPK,* some potential functions of *GuMAPK* genes were preliminarily inferred. The results revealed that *AtMPK6*, which is homologous to *GuMAPK6* and 6*, interacts with MKK1 to mediate ABA (abscisic acid)-dependent CAT1 expression in response to drought and salt stress. Additionally, *AtMPK3* (homologous to *GuMAPK3* and *3-2*) and *AtMPK6* (homologous to *GuMAPK6* and 6*) interact with PR1. *AtMPK12* (homologous to *GuMAPK12* and *12-2*) interacts with respiratory burst oxidase homolog D (RBOHD). *AtWRKY33* is involved in plant defense responses against fungal pathogens, and F23F1.6 acts as a protein phosphatase, exerting a negative influence on defense responses. Stress and defense signaling involve the participation of deactivated MPK4 and MPK6 MAP kinases. Proteins such as GuMAPK3/3-2/6/6*/16/16-2/5/5-2 in *G. uralensis*, which are homologous to *A. thaliana* MAPKs, are speculated to have similar functions.

### 3.8 Construction of the *GuMAPK16-2* overexpression vector and subcellular localization

**FIGURE 10:**
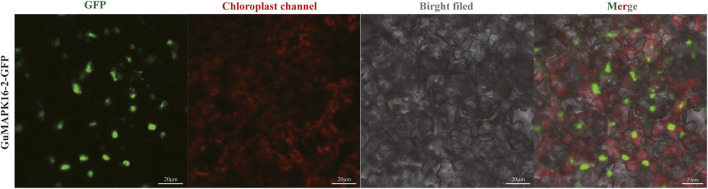
Subcellular localization of *GuMAPK16-2*. The green fluorescence is GFP labeled *GuMAPK16-2*.

Subcellular localization of the *GuMAPK* gene family has not been reported to date. On the basis of the gene expression profiles under salt stress and *B*. *subtilis* treatment, we selected a gene significantly expressed under high-concentration stress (*GuMAPK16-2*) to validate the subcellular localization of the MAPK genes in *G. uralensis*. The pC1300-GFP-*GuMAPK16-2* fusion expression vector was constructed and transformed into *Agrobacterium tumefaciens* competent cells, followed by transient transfection into *Nicotiana benthamiana* leaves. Observation was conducted using confocal fluorescence microscopy, where the target gene linked with the GFP exhibited green fluorescence, whereas chlorophyll emitted red fluorescence under excitation light at approximately 640 nm. The green fluorescence signal of the *GuMAPK16-2*-GFP fusion protein was predominantly distributed in the cell nucleus, indicating that the *GuMAPK16-2* gene is likely located in the nucleus. This finding further validates our earlier prediction regarding the subcellular localization of this gene (Figure 10).

## 4 Discussion

The MAPK cascade is a widely conserved functional module widely present in eukaryotes and has been extensively studied in model organisms such as *A. thaliana*, *Oryza sativa L.*, and other plants (Zhang et al., 2018). During plant growth and development, the MAPK cascade transmits extracellular stimuli to the nucleus, predominantly regulating cellular processes, including proliferation, differentiation, and apoptosis, by modulating gene expression (Kosová et al., 2011). As members of the protein kinase superfamily, MAPKs catalyze the phosphorylation of target proteins, thereby modifying their activity, binding affinity, stability, or cellular localization. Numerous investigations have demonstrated the pivotal involvement of MAPK transcription factors in addressing both biotic and abiotic stresses (Liu et al., 2013; Baillo et al., 2019).

The characteristics and functions of the MAPK gene family have been identified and studied in various plants, such as *A. thaliana*, *O. sativa L.*, *Zea mays L*. (Wu et al., 2014), and *S. lycopersicum* (Yao et al., 2022). Through multiple sequence alignments of *GuMAPK* proteins and phylogenetic analysis with Arabidopsis, the 21 *GuMAPKs* were classified into four subgroups: A, B, C, and D. Subgroups A, B, and C contain the TEY activation loop, whereas subgroup D contains the TDY activation loop (López-Bucio et al., 2014). The prevalence of the TEY motif in MAPK gene family members is consistent with that in *A. thaliana*, suggesting a potentially critical role for TEY-type MAPK genes in the evolution of dicotyledonous plants. Across all studied species, *MAPK3* and *MAPK6* within subgroup A show homology, either both present or both absent, underscoring their essential functions in plant growth, development, and responses to biotic and abiotic stresses, as validated across diverse plant species (Sinha et al., 2011; Raina et al., 2012; Mohanta et al., 2015). Additionally, *MAPK10* in subgroup A was identified only in cruciferous plants, with the exception of *A. thaliana*, suggesting its conservation within this family but loss in other plant families during evolution (Wang et al., 2022). Similarly, *MAPK11* in subgroup B shows a similar pattern except it is also lost in *A. thaliana*. In *G. uralensis*, subgroup C contains only homologs of *MAPK1* and *MAPK7*, which is consistent with the findings in lettuce (Cannon et al., 2004) and tomato, indicating closer evolutionary relationships among them. Subgroup D members are the most unique among the MAPK gene family members and were identified in all studied plants (Meng and Zhang, 2013). Collinearity analysis offers insights into the potential evolutionary mechanisms of the *G. uralensis* gene family, highlighting the critical role of gene duplication—including tandem and segmental duplication—in genome expansion, rearrangement, functional diversification, and the formation of extensive gene families. Within *GuMAPKs*, the identification of three pairs of homologous genes involved in segmental duplication suggests that this process serves as a primary mechanism driving evolutionary changes (Wang et al., 2021). Moreover, the presence of 11, 11, and 14 pairs of homologous genes shared between licorice and *A. thaliana*, tomato, and cucumber, respectively, also indicates closer evolutionary relationships between them.

Gene structure plays a crucial role in determining gene expression and function, with distinct characteristic motifs observed among different plants indicating their evolutionary relationships and functional divergence. (Mohanta et al., 2015). In the analysis of conserved motifs, *GuMAPKs* from different subgroups exhibited both intragroup similarity and intergroup differences in motif sequences. Except for *GuMAPK3-2*, all *GuMAPKs* present highly conserved motif sequences, among which motif 6 represents the T-loop, a unique activation loop in the MAPK family. Activation of downstream MAPKs occurs through phosphorylation of the TDY or TEY motifs within their activation loop. This phosphorylation event leads to the subsequent phosphorylation of various downstream substrates, thereby regulating gene expression. The distribution of introns and exons in *GuMAPK* gene structures mirrors this characteristic pattern, demonstrating consistency within different groups while also revealing notable differences both within and between these groups (Wei et al., 2021; Alabd et al., 2023). For example, except for *GuMAPK3-2*, members of subgroup D exhibit the most complex gene structures, markedly more than those in other groups. Comparable structural motifs have been detected in various plant species, exhibiting substantial conservation within specific groups and increased divergence between these groups. For example, in lettuce MAPK gene families, there is a significant disparity in the number of introns and exons between subgroup D and subgroups A, B, and C, which are typically characterized by subgroup D harboring a greater abundance of both introns and exons (Wei et al., 2021).

The promoter regions of *GuMAPKs* contain numerous *cis*-acting elements, such as MYB elements, ABRE (ABA-responsive element), MYC elements, the CGTCA motif, and the TGACG motif (MeJA-responsive element), indicating the widespread involvement of *GuMAPKs* in the light response and biotic and abiotic stress processes of *G. uralensis*. In addition to the G-box, the most frequently occurring element is the ABA-responsive element. The ABRE element (PyACGTGGC) is an important *cis*-acting element involved in ABA-regulated gene expression, and it has been shown to respond to stresses such as low temperature, drought, and high salinity in both rice and *A. thaliana* (Han et al., 2013). Notably, *GuMAPK6* and 6* have completely different promoter sequences, as well as differences in cis-acting elements, which could lead to functional differences between the two genes. But under certain conditions, the functions of the two genes are also highly likely to overlap. To counteract the adverse effects of environmental stresses, plants have evolved adaptive mechanisms or specific growth habits to avoid stress, and phosphorylation can participate in fine-tuning to cope with abiotic stress. Therefore, analysis of phosphorylation sites is crucial for understanding the mechanism of action of *GuMAPK* protein kinases. In addition to specific phosphorylation sites, phosphorylation by protein kinase C (Phos-PKC) and casein kinase II (phso-CKII) occurs most frequently. Professor Lieven De Veylder (Huang et al., 2021) reported that the plant casein kinase CKII regulates root growth under aluminum toxicity and phosphorus deficiency conditions mediated by SOG1 in the DNA damage response, revealing the significant role of protein phosphorylation in plant growth and development as well as in response to abiotic stress signals. The three-dimensional structure of proteins determines how they interact with other molecules and dictates their roles within cells. The diverse three-dimensional structures of proteins enable them to perform a wide range of functions in cells, and the protein’s three-dimensional structure can be inferred from its amino acid sequence. Three-dimensional structure prediction of *GuMAPKs* revealed that α-helices and irregular coils are the main spatial structures of *GuMAPKs* and that stable spatial conformations are the basis for protein function. Furthermore, the GuMAPK gene family consists of similar structural domains, with highly similar predicted three-dimensional structures within the same subgroup. These predictions align with the characteristic features of the gene family proteins and exhibit comparable conserved domains.

Numerous studies have underscored the extensive engagement of the plant MAPK cascade signaling pathway in diverse responses to both abiotic and biotic stresses (Liu et al., 2013). Among the notable MAPKs in *A. thaliana* are *AtMPK3* and *AtMPK6*. Protein‒protein interaction network analysis revealed that *AtMPK3* (homologous to *GuMAPK3* and *3-2*) and *AtMPK6* (homologous to *GuMAPK6* and 6*) interact with *PR1*. PR1 is a plant pathogenesis-related protein that belongs to a widely distributed protein superfamily. It was initially identified as a protein strongly induced under both biotic and abiotic stress conditions. The overexpression of PR1 in immune defense mechanisms enhances resistance against pathogens (Pitzschke et al., 2009). *AtMPK12* (homologous to *GuMAPK12* and *12-2*) interacts with RBOHD. *RBOHD* is a calcium-dependent NADPH oxidase that generates superoxide, which participates in ROS production during pathogen incompatibility interactions and in ROS-dependent signaling during UV-B and abscisic acid treatment (Yamada et al., 2016). In addition, genes such as *VSP2, MKS1, ANP1, MKK1*, and *MKK4* interact with MAPKs, all of which are key genes identified in previous studies in which *B. subtilis* was inoculated to counteract salt stress in *G. uralensis*, indicating that homologous *GuMAPKs* also have similar functions. Notably, *GuMAPK16* and *16-2*, which are homologous to *AtMPK16*, interact with *MKK1* (MAPK kinase 1) and *MKK4* (MAPK kinase 4). Andrea Pitzschke et al. reported that the MAPK kinase *MKK1* is part of the cascade responsible for regulating the accumulation of ROS and salicylic acid (SA) (Egamberdieva et al., 2017). According to several reports, most of the transcription factors highly responsive to various ROS-induced conditions are regulated by *MEKK1,* mainly through the *MEKK1-MKK1/2-MPK4* pathway. Yamada K et al. reported that the *MAPKKK5-MKK4/5-MPK3/6* pathway plays an important role in chitin signal transduction in *A. thaliana* (Savojardo et al., 2018).

On the basis of the transcriptome data of 21 *GuMAPKs* expressed in response to salt stress in *G. uralensis* after inoculation with *B*. *subtilis* obtained from previous studies by our research group, at a salt concentration of 200 mmol/L, the expression of *GuMAPK5* (*P* = 0.048), *GuMAPK7* (*P* = 0.047), *GuMAPK9* (*P* = 0.0004), *GuMAPK16* (*P* = 0.048), and *GuMAPK20-2/20-3* (*P* = 0.029) was significantly upregulated, indicating their response to salt stress in *G. uralensis*, possibly playing a more crucial role in this process. However, after inoculation with *B*. *subtilis*, the expression of *GuMAPK5* (*P* = 0.021), *GuMAPK7* (*P* = 0.033), and *GuMAPK16* (*P* = 0.007) was significantly upregulated, suggesting that *B*. *subtilis* enhanced the tolerance of these genes to saline‒alkali conditions, thereby alleviating the impact of salt stress on the quality of *G. uralensis*. Notably, at a high salt concentration of 300 mmol/L, *GuMAPK16-2* (*P* = 0.038) was the only gene whose expression significantly differed, and after inoculation with *B*. *subtilis*, the expression of *GuMAPK16-2* (*P* = 0.030) was also significantly downregulated. These findings provide insights into the molecular mechanism by which *B. subtilis* inoculation affects the response of the MAPK signaling pathway to high salt stress in *G. uralensis*.

## 5 Conclusion

This study conducted a systematic analysis of the MAPK gene family in *G. uralensis*, identifying 21 *GuMAPK* genes in the genome. Various aspects of these genes, including their physicochemical properties, gene structures, conserved motifs, chromosomal locations, *cis*-acting elements, and phosphorylation sites, were thoroughly examined using bioinformatics methods. This study also investigated the response of *GuMAPK* genes to salt stress and *B*. *subtilis* inoculation, highlighting *GuMAPK16-2* as a key gene within the *G. uralensis* MAPK gene family that responds to high-salt stress under *B*. *subtilis* inoculation. Subcellular localization validation was conducted, followed by the establishment of a protein‒protein interaction network that identified multiple MAPK genes involved in regulating gene expression processes under salt stress conditions. These findings provide a theoretical basis for understanding the mechanisms underlying salt stress resistance in *G. uralensis* and present an opportunity for further exploration of the evolutionary trajectory and functional roles of the *GuMAPK* gene family in response to various stressors, both biotic and abiotic.

## Data Availability

The datasets presented in this study can be found in online repositories. The names of the repository/repositories and accession number(s) can be found in the article/Supplementary Material.

## Abbreviations

*G*. *uralensis*, *Glycyrrhiza uralensis* Fisch; *B*. *subtilis*; Bs, *Bacillus subtilis*; ROS, Reactive oxygen species; pI, Isoelectric point; GRAVY, Grand average of hydropathicity; SA, Salicylic acid; ABRE element, ABA-Responsive Element; CDS, Coding sequence; ABA, Abscisic acid; MAPKK, MAPK kinase; MAPKKK, MAPKK kinase; CD domain, Common Docking Domain; RBOHD, Respiratory Burst Oxidase Homolog D.
