# Supplementary figures and images for: Genome-wide identification of Glycyrrhiza uralensis Fisch. MAPK gene family and expression analysis under salt stress relieved by Bacillus subtilis
Source: Front Genet. 2024 Jul 26;15:1442277. doi: 10.3389/fgene.2024.1442277 (PMC11310058; doi:10.3389/fgene.2024.1442277)

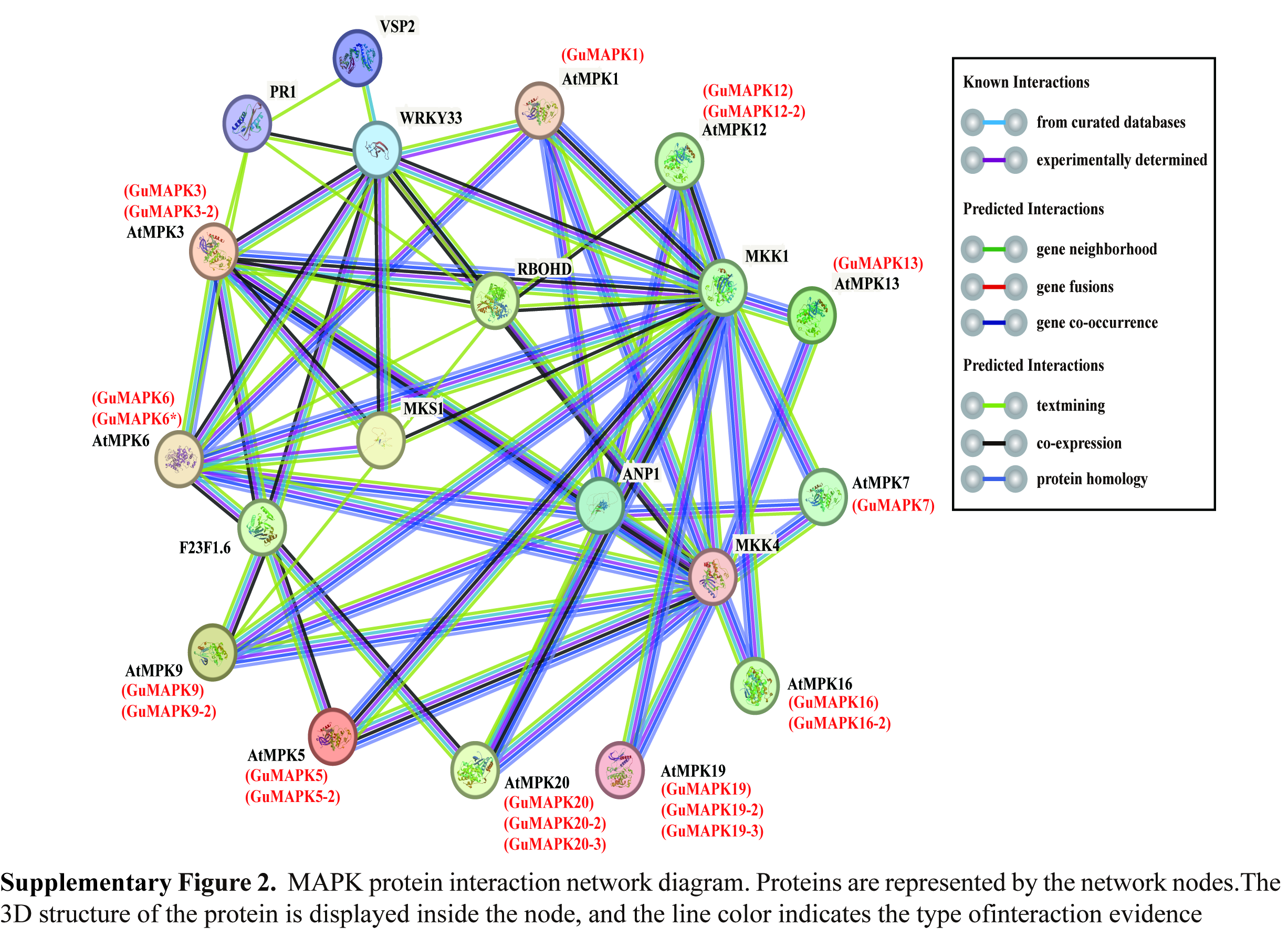

Supplement: Supplementary file 3 [file Image2.TIF]

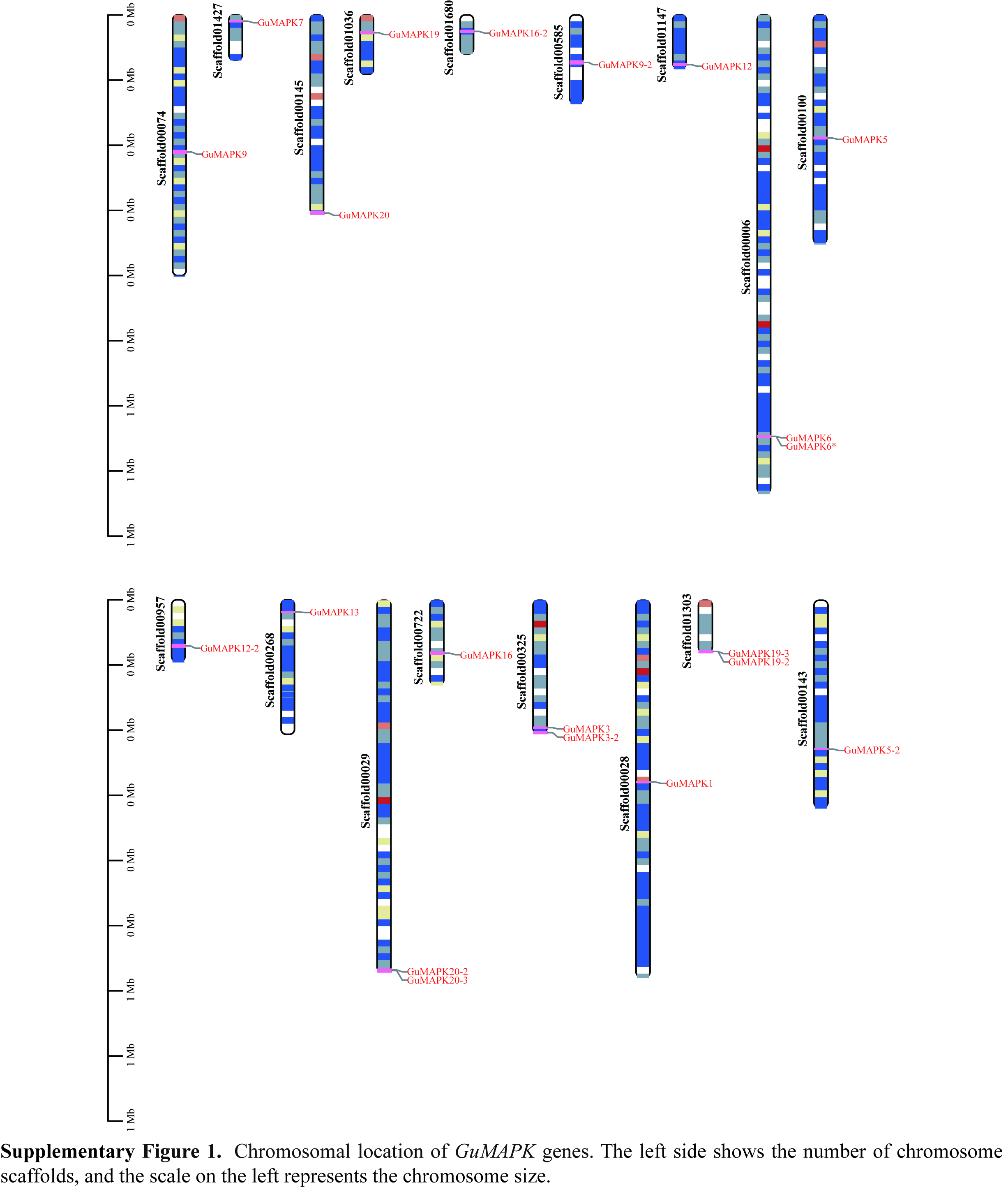

Supplement: Supplementary file 4 [file Image1.TIF]
